# Evaluation of superficial femoral artery-lesions after percutaneous transluminal angioplasty: color-coded summation images vs. monochromatic digital subtraction angiography

**DOI:** 10.1186/s12880-020-00468-1

**Published:** 2020-06-18

**Authors:** Anne Marie Augustin, Irina Thein, Nicole Rickert, Thorsten Klink, Thorsten Alexander Bley, Ralph Kickuth

**Affiliations:** 1 Department of Diagnostic and Interventional Radiology, University Hospital Würzburg, Würzburg, Germany; 2 Department of Diagnostic and Interventional Radiology, Hospital Bayreuth GmbH, Bayreuth, Germany

**Keywords:** Angiography, Angioplasty, Diagnostic imaging, Digital subtraction angiography, Endovascular procedures, Interventional radiology, Imaging analysis, Imaging postprocessing, Parametric color-coding, Peripheral arterial occlusive disease, Vascular imaging

## Abstract

**Background:**

Percutaneous transluminal angioplasty (PTA) is increasingly requested in the therapy of peripheral arterial occlusive disease. The evaluation of the technical result after balloon angioplasty with regard to bailout stenting is highly dependent on the operators´ subjective assessment and mainly based on the monochromatic digital subtraction angiography (DSA) images. The aim of this study was to compare color-coded single image as a novel diagnostic tool with monochromatic DSA for the analysis of flow limitation and need for stent implantation after PTA of superficial femoral artery (SFA) stenoses.

**Methods:**

During a period of 18 months, 213 SFA lesions were treated by PTA with a standard balloon in 170 patients, resulting in a total of 193 endovascular procedures. The median age of the patients was 77 years (range, 35–96 years). Median length of the treated lesions was 10.5 cm (range, 1.0–50 cm). Three interventional radiologists retrospectively evaluated the results of balloon angioplasty with monochromatic as well as post-processed color-coded DSA images for flow limitations to decide if subsequent stent implantation was necessary. Consensus reading of two experienced interventional radiologists 2 months after the initial review served as reference standard to perform a receiver operating characteristics (ROC) analysis.

**Results:**

ROC analysis for readers A, B and C showed area under the curve (AUC) values of 0.797, 0.865 and 0.804 for color-coded DSA and AUC values of 0.792, 0.843 and 0.872 for monochromatic DSA: a significant advantage of color-coded over conventional monochromatic DSA was not found for readers A and B (*p* > 0.05). Results of reader C were significantly better in the assessment of monochromatic images (*p* = 0.023). Diagnostic confidence using color-coded images was slightly higher than in monochromatic images (κ = 0.486 vs. κ = 0.459).

**Conclusions:**

In this study, color coded DSA did not reveal to be superior to conventional monochromatic DSA when evaluating results of PTA and when deciding whether stent implantation is necessary or not. This technology, however, requires further experiences with special regard to homogeneously trained radiologists and to the time requirement.

## Background

Peripheral arterial occlusive disease (PAOD), usually secondary to artherosclerotic alterations, is a medical condition with increasing incidence and major impacts on the patients´ morbidity and mortality [1, 2]. Percutaneous endovascular treatment of PAOD plays an important role in the therapeutic portfolio and offers advantages, especially in multimorbid or elderly patients [3]. With digital subtraction angiography (DSA) and revascularization techniques - like percutaneous transluminal angioplasty (PTA) and stent implantation - endovascular interventions combine both diagnostic and therapeutic intentions and represent the first-line therapy in a growing number of cases. The most common initial endovascular treatment approach in arterial occlusions and stenoses of the femoropopliteal arteries is plain old balloon angioplasty (POBA). If results are not satisfying in terms of bailout situations, prolonged POBA or stent implantation are the options usually applied subsequently [4]. Since conventional monochromatic DSA does not offer quantification features except for two-dimensional measurement of the vessel lumen diameter, assessment of a stenosis is highly dependent on the experience of the interventional radiologist performing the procedure.

As a novel and potentially useful tool for assessment of information gained during DSA, the beneficial value of color-coded DSA had already been proofed in the evaluation of carotid cavernous-fistulas as well as cerebral arteriovenous malformations [5–7]. In this context, authors emphasize the fast availability as much as the acquisition of quantitative hemodynamic parameters as main advantages. Moreover, periprocedural usage of this technique might support therapeutic monitoring in order to evaluate therapy effects [8]. Concerning endovascular management of the lower extremities, one study revealed a high correlation of the changes in color-coded DSA parameters and pre- and postinterventional performed ABI, suggesting this method as adequate for the evaluation of intervention outcomes [9]. In contrast to that, a study of Kostrzewa et al. did not reveal strong correlations between changes in time-density curves created by color-coded software and clinical parameters like ABI and ultrasound peak systolic velocity ratio (PSVR) [10]. These parameters were considered to be independent and to rate color-coded DSA as beneficial for technical outcome control in treatment of superficial femoral artery (SFA) and popliteal artery. Reekers et al. utilized a similar post-processing software to visualize tissue perfusion represented by time-density curves of contrast volume. With an increase of volume flow to the foot, perfusion angiography showed a benefit in the success-evaluation of revascularization in critical ischemia of the lower limb [11]. To date, only little data exist addressing the value of this post-processing method in the assessment of flow-limitations during an endovascular peripheral artery disease (PAD) intervention. The aim of this study was to evaluate the usefulness of color-coded single image in comparison with monochromatic DSA for assessment of flow limitation and need for stent implantation after PTA of SFA stenoses.

### Materials

#### Study sample and scheme

A retrospective review of the archives of our interventional radiology division between April 2014 and October 2015 yielded the cases of 170 patients who consecutively underwent PTA with a standard balloon in 213 PAD lesions of the SFA with a total of 193 endovascular procedures. The median age of the patients was 77 years, ranging between 35 and 96 years. The clinical characteristics of our study population referring to the procedures are summarized in Table 1.

**Table 1:** Clinical characteristics of the patients referring to the procedures

Clinical characteristics	number	%
Sex		
Male	100	51.8
Female	93	48.2
Fontaine classification [12]		
2a	9	4.7
2b	46	23.8
3	18	9.3
4	108	56.0
unknown	12	6.2
Cardiovascular risk factors		
Smoking history	67	34.7
Diabetes mellitus	87	45.1
Renal failure	143	74.1
Overweight	102	52.9
Arterial hypertension	159	82.4
Dyslipidaemia	67	34.7

Patient-related exclusion criteria for the procedure included history of anaphylactic reaction to contrast, pregnancy, untreated inflow disease of the ipsilateral iliac artery, coexisting aneurysmatic disease of the abdominal aorta, iliac or popliteal artery, significant gastrointestinal bleeding and uncorrectable coagulopathy. Patients were not systematically evaluated for inflammatory vasculitis. Following local anesthesia in all patients, endovascular procedures were performed via an ipsilateral antegrade transfemoral vascular access. Monochromatic (black-white modus) DSA source image acquisition were gained using an Axiom Artis Zee Angio suite (Siemens, Forchheim, Germany). Treatment side was the right SFA in 107 and the left SFA in 106 of the 213 PAD lesions. Median length of lesion was 10.5 cm, ranging from 1.0 to 50 cm. Evaluation of patients´ medical records revealed existing clinical follow-up examinations in 73.7% of the cases. Median follow-up time was 4.5 months and ranged between 0.5 and 80 months. Based on the available medical records and patients´ charts, a Kaplan-Meier analysis resulted in a primary clinical patency rate (defined as maintenance of improvement in one clinical category) of 67.0% after 6 months and of 54.7% after 12 months, respectively.

All patients were examined as part of routine care and gave informed consent for PTA. The local institutional review board waived its approval for this retrospective postprocessing study (Waiver No. 20160514 01).

#### Post-processing

**Fig. 1:**
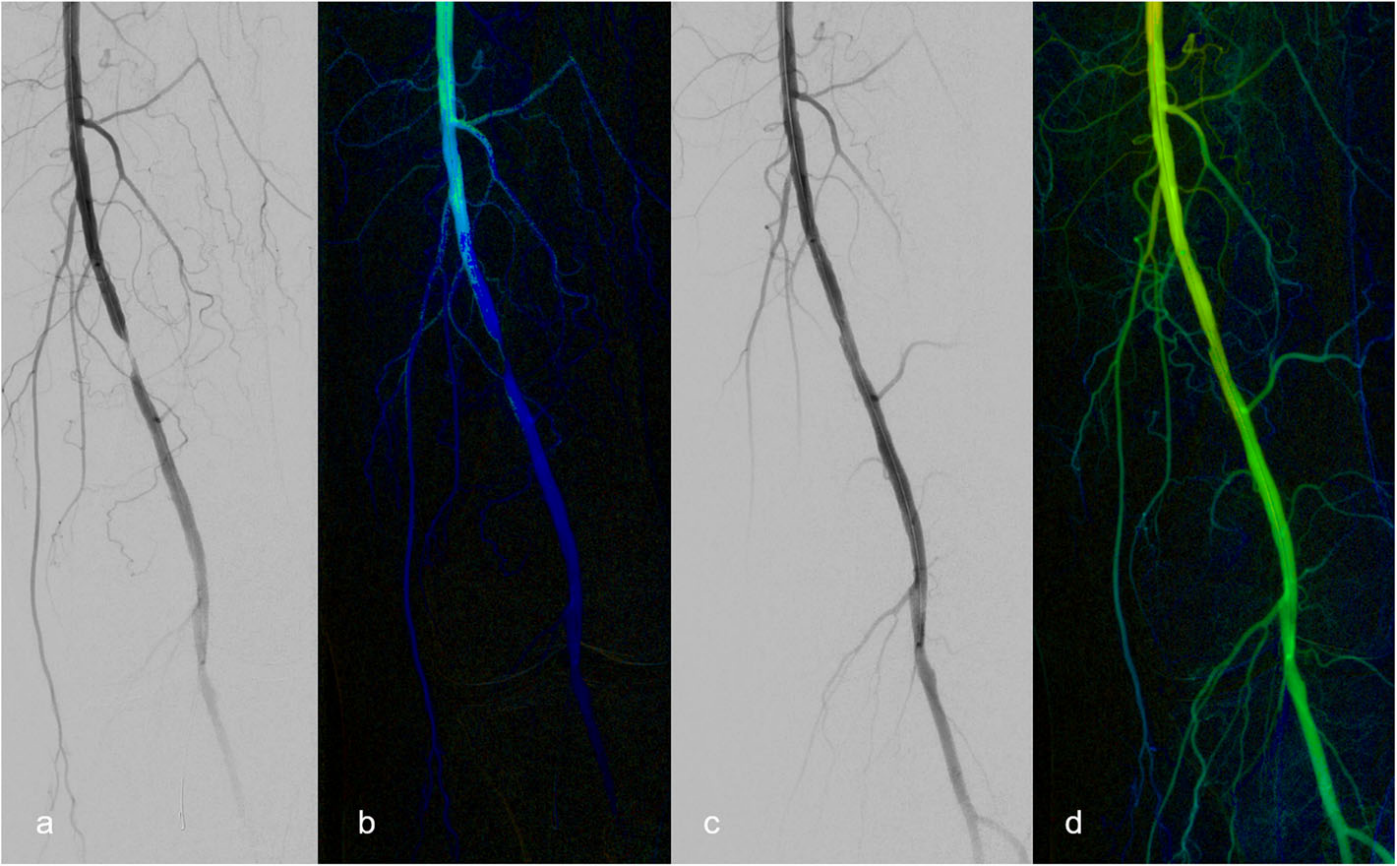
Monochromatic digital subtraction angiography (DSA) with corresponding color-coded DSA image of a patient before and after percutaneous transluminal angioplasty (PTA). a Initial angiography demonstrates high grade stenosis of the distal superficial femoral artery (SFA). b The corresponding color-coded image shows reduced blood flow due to stenotic lesion, expressed by a color change of intraluminal contrast from bright green to dark blue. c, d Improved vessel diameter after PTA is demonstrated by enhanced blood flow, expressed by homogenous green color. All three readers concluded that no stenting was necessary for both the DSA series and the colored image

**Fig. 2:**
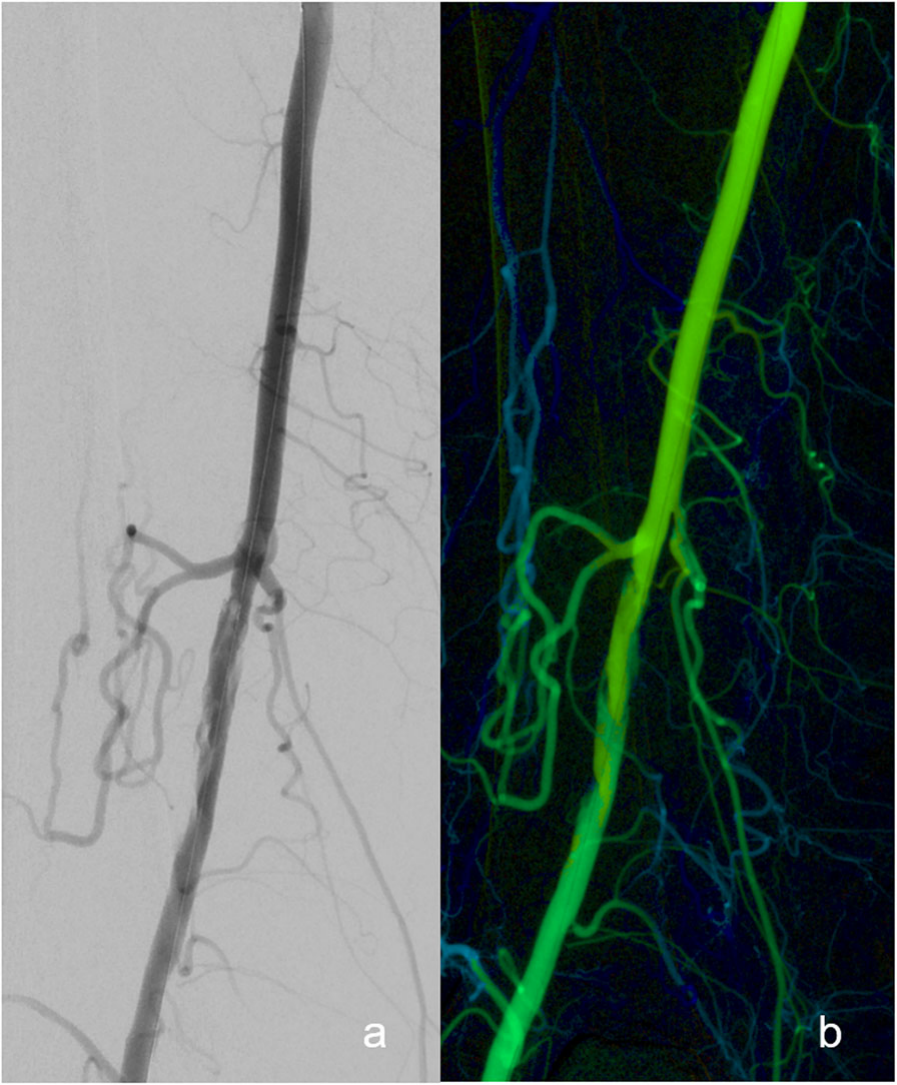
a Conventional digital subtraction angiography (DSA) with corresponding color-coded DSA image of a patient after percutaneous transluminal angioplasty (PTA) of the SFA displays a dissection. b Correlating color-coded DSA image revealed no relevant limitation of blood flow

DSA images were sent to the local picture archiving and communication system (PACS)® (Syngo Plaza, Siemens Healthcare GmbH, Erlangen, Germany) and post-processed at a specialized working station (Syngo XWP, Siemens Healthcare GmbH, Erlangen, Germany) with system software WinNT 5.2, SP 2. Parametric color-coded pictures were generated using the commercially available software Syngo iFlow® (Siemens Healthcare GmbH, Erlangen, Germany). This novel approach enables the visualization of DSA series in a single picture with color coding of blood flow curves depending on the velocity of single pixel markers. Therefore, the color-coding algorithm measures the time between contrast agent injection and the maximum opacification for each pixel. In this regard, dark blue indicates a very slow blood flow and red represents a very high flow velocity (Figs. 1 and 2). Moreover, other flow parameters such as the area under the curve can be acquired for any region of interest, leading to a more precise characterization of the inflow and outflow situation.

#### Evaluation of imaging

Three interventional radiologists with 19 (Reader A), four (Reader B) and 2 years of experience (Reader C) independently assessed the diagnostic power of conventional monochromatic DSA series and color-coded summation images to decide whether residual stenosis, elastic recoiling, or dissection post PTA should be considered as flow-limiting and therefore stent implantation after POBA should have been performed. Reader C was completely unfamiliar with the color-coded technology. Before their first interpretation, radiologists did not have a training session. With a number of 213 PAD lesions and the use of monochromatic and color-coded DSA this resulted in a total of 1278 observations (639 per DSA method). Monochromatic series and color-coded images were presented in a random order and in two separate sessions at least 7 days apart to avoid observer bias. Furthermore, the readers were blinded to clinical data and outcome of the IR procedure. The time allowed for interpretation of the images was not restricted. Diagnostic confidence in the reader’s decision-making was rated on a 5-point ranking scale for both imaging techniques, as shown in Table 2.

**Table 2:** Scale of evaluation options

1	No stent implantation necessary
2	Presumably no stent implantation necessary
3	Uncertain
4	Presumably stent implantation necessary
5	Stent implantation necessary

A consensus reading of the monochromatic and color-coded images was performed two months after the initial review by two experienced interventional radiologists and served as reference standard. This consensus resulted in 91 lesions not requiring stent implantation. In 122 lesions consensus evaluation showed the necessity of stent implantation due to blood flow limitation with corresponding color gradients in the color-coded images. The underlying lesion characteristics leading to the decisions while evaluating the monochromatic images are presented in Table 3.

**Table 3:** Consensus reading results of 213 superficial femoral artery lesions after previously performed percutaneous endoluminal balloon angioplasty

Residual stenosis	29
Dissection	39
Elastic recoiling	1
Residual stenosis + dissection	31
Residual stenosis + elastic recoiling	5
Residual stenosis + dissection + elastic recoiling	14
Dissection + recoiling	3
No reason for stent implantation	91
Total	213

### Statistical analysis

Data were collected with Excel (Microsoft Office 365 ProPlus, Version 1803). Statistical analysis was performed using a dedicated software (MedCalc, Version 6.00.014). Diagnostic accuracy was determined by Receiver Operating Characteristics (ROC curves) and the area under the ROC curve (AUC). For ROC analysis, sensitivity and specificity (in percent) of a test are displayed in a diagram. With values between 0.5 and 1, the area under the curve represents the precision of a test in one number. Student’s t-test was used to evaluate differences in the AUC. *P*-values less than 0.05 were considered statistically significant. The reference standard was defined by the results of a second reading of all images by the two most experienced readers in consensus 2 months after the initial review. Based on a Youden-Index analysis, category 1 or 2 indicated no stent implantation, whereas categories 3, 4 or 5 indicated stent implantation. Inter-observer reliability between the three radiologists was evaluated by using two types of kappa (κ) statistics. Additional to unweighted kappa, kappa analysis weighed by the three possible decisions (no stent, not sure, stent) was performed. A kappa value < 0.4 was rated as poor reliability, whereas a kappa value between 0.41–0.75 indicated good agreement and kappa values < 0.75 was rated as highest congruence. For quantification of diagnostic confidence, numerical answers were evaluated in three categories. In this regard, answer 1 and 5 were rated as strong diagnostic confidence, answer 2 and 4 were rated as slightly uncertain and answer 3 was rated as moderate diagnostic confidence.

## Results

In 101 of the 213 hemodynamically relevant stenoses and occlusions of the SFA further stent implantation was performed following PTA. In the other 112 cases, only PTA was performed with a satisfactory result requiring no further therapy.

**Fig. 3:**
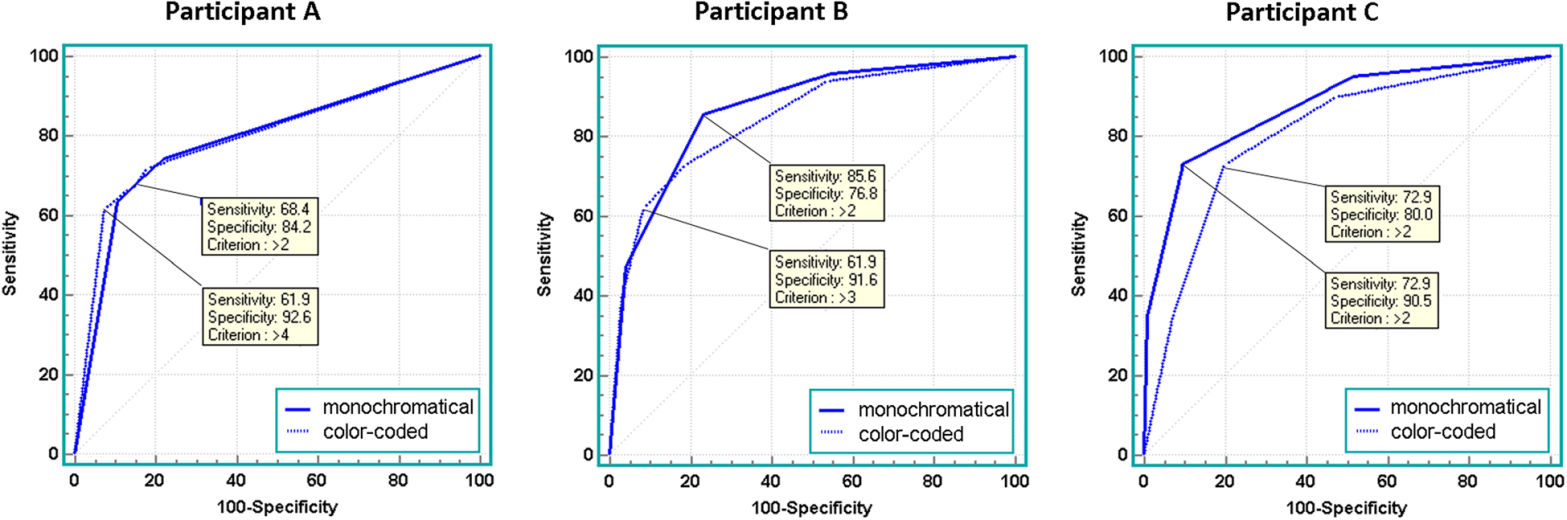
Composite receiver operating characteristic (ROC) curves of the three readers’s evaluation of monochromatic and color-coded DSA-images

Results of Reader A revealed no significant difference between AUC of monochromatic DSA (0.792) and color-coded DSA (0.797) *p* = 0.890, resulting in nearly parallel ROC-curves (Fig. 3). Monochromatic DSA showed sensitivity and specificity values of 56.4 and 90.5%, and polychromatic images values of 61.9 and 90.5%, respectively. Results of Reader B revealed no significant difference between AUC of monochromatic DSA (0.843) and color-coded DSA (0.865) *p* = 0.453. Monochromatic DSA showed sensitivity and specificity values of 61,9% and 91,6%, respectively. Reader C performed with a significant greater AUC using conventional monochromatic DSA as compared to color-coded DSA (0.872 vs. 0.804, *p* = 0.023). Sensitivity was 72.9% in both monochromatic and color-coded images and specificity was 90.5% in monochromatic and 80.0% in color-coded images.

Mean sensitivity and specificity of all three readers were 75.7 and 83.8% for monochromatic images and 70.9 and 82.1% for color-coded images, respectively. Summarized results for a cut-off > 2 based on the Youden-Index are shown in Table 4.

**Table 4:** Results of ROC-analysis based on a cut-off-value > 2, as well as percentages of decisions concordant to consensus

Participants		monochromatic	color-coded	difference	*p*-value
A	Sensitivity	0.686	0.669		
	Specificity	0.842	0.85		
	PPV	0.844	0.853		
	NPV	0.684	0.675		
	AUC	0.792	0.797	0.005	0.890
	Decision concordant to consensus	0.737	0.728		
B	Sensitivity	0.856	0.729		
	Specificity	0.768	0.811		
	PPV	0.821	0.827		
	NPV	0.811	0.706		
	AUC	0.865	0.843	0.022	0.453
	Decision concordant to consensus	0.714	0.744		
C	Sensitivity	0.729	0.729		
	Specificity	0.905	0.800		
	PPV	0.905	0.819		
	NPV	0.729	0.704		
	AUC	0.872	0.804	0.068	0.023
	Decision concordant to consensus	0.803	0.761		
A + B + C	Sensitivity	0.757	0.709		
	Specificity	0.839	0.821		
	PPV	0.857	0.832		
	NPV	0.741	0.695		
	Decision concordant to consensus	0.750	0.744		

With a κ = 0.258 for monochromatic series and κ = 0.246 for color-coded images, weighted kappa (κ) statistical analysis revealed poor inter-observer reliability for both imaging techniques. Weighted and adjusted κ summarizing decision 1 + 2 and 4 + 5 resulted in κ of 0.459 for monochromatic and κ of 0.486 for color-coded images, which means a moderate inter-observer agreement for both imaging techniques with only slightly better reliability for post-processed quantitative images. All κ-values include the results of three observers.

Overall, in 52.9% of cases, evaluation of conventional and color-coded images yielded the same result whether stent implantation is necessary or not (Reader A 58.7%, Reader B 44.7%, Reader C 55.4%). Evaluation of color-coded images led to a different result with regard to stent implantation in 19.2% of cases.

Referring to the consensus, right decision was made in a total of 75.0% of the cases when evaluating of the monochromatic images and in 74.4% of assessing the color-coded images. The results determined for each participant are also shown in Table 3.

**Fig. 4:**
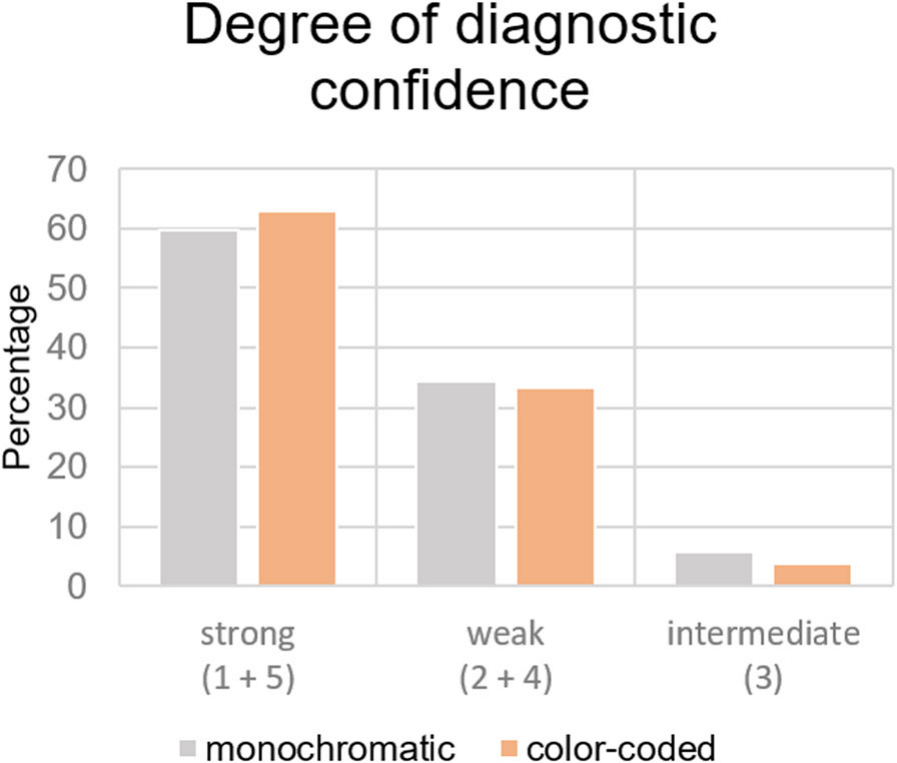
Quantification of certainty in the evaluation of monochromatic and color-coded images.

No significant differences of observer’s diagnostic confidence in the decision-making process between both imaging techniques was found. Only little advantage was shown in evaluation of color-coded DSA, with a slightly increased amount of answers rated as certain (answer 1 and 5) (Fig. 4).

## Discussion

In the evaluation of PAOD, DSA represents the obvious diagnostic approach, when endovascular therapy is contemplated. Furthermore, DSA is necessary during an endovascular intervention in order to evaluate any flow-limitation after POBA. Nevertheless, the visual assessment of stenotic lesions in the peripheral arterial system based on two-dimensional, monochromatic DSA images only remains subjective and carries the risk of misjudgment with frequent overestimation of stenosis [13].

As one result of striving for increased information extraction of monochromatic DSA series, parametric color-coded DSA image post-processing algorithms are available from different providers. These tools enable color-supported quantification and visualization of hemodynamic information like blood flow velocity, summarized in one picture. Parameters like time to peak (TTP) and peak opacification profiles support analysis of hemodynamic characteristics before and after an intervention.

The use of this software has been tested mainly in the field of neuroradiology [5–7, 14]. In this study, the utilization of color-coded DSA in the assessment of post PTA results of peripheral vessels in comparison with conventional DSA was evaluated. In this context, results could show an impact on further clinical practice with a recommended implementation of color-coded DSA in the clinical routine. Consensus reading was used as reference standard in order to increase the degree of objectivity. Furthermore, in order to avoid the impact of different lesion locations and thus deviations in hemodynamics, only lesions of the SFA were included. To reduce bias, evaluation of conventional monochromatic and post-processed polychromatic images was achieved by separated interpretation sessions. In addition, to prevent the risk of remembering the cases and thus interference with the consensus results, imaging evaluation and consensus building was performed at an interval of at least 2 months.

In a direct comparison, our results did not show superiority of color-coded DSA single images over conventional monochromatic DSA. In fact, one reader (C) showed significant better results in evaluation of conventional monochromatic DSA-images with 80.3% of right decisions when evaluating conventional images and 76.1% when evaluating color-coded images. For the other two radiologists, no significant difference was revealed when reading the two image-techniques. Referring to reader C, a major reason for the better results in favor of monochromatic DSA imaging might be that he was unfamiliar with color-coded DSA images prior to the study and that he was the least experienced radiologist in the study. As a consequence, he might have felt more comfortable with the gray scale images. However, it may need time for less experienced radiologists to get used to color scale data. So based on a cross sectional study alone no definite conclusion can be made. The more eye training radiologists get and with more experience, they may find it more useful.

Utilization of color-coded DSA imaging tools was predefined in the study setup. Additional software applications such as time-intensity curves, time-to-peak and area under the curve parameter were not allowed to use. On the one hand, this leads to more stable study conditions, but on the other hand, it also carries risk of not utilizing the full potential of color-coded DSA and thus, results for color-coded images might have been degraded. Moreover, only single images were available in the evaluation of post-processed color-coded images, while conventional DSA were presented as series of images. Under this aspect, it seems notable that both imaging techniques revealed similar results.

Despite our results, there are some obvious benefits of color-coded images: Color-coded DSA offers instant information of blood flow in a single image and thus allows fast reading, suggesting that this technique is suitable for assessment during an intervention. Furthermore, color-coding of conventional DSA imaging does not necessitate further radiation exposure or contrast agent since color-coded images are created using the monochromatic DSA images already available.

This study has several limitations. First, the results of color-coded DSA images are not free of procedure-correlated influences. Ionita et al. [15] revealed the high dependence of results of color-coded DSA on contrast injection parameters, like catheter position as well as injection volumes and rates. They concluded that utilization of postprocessed parametric images should be limited to the evaluation of blood flow before and after treatment in the same patient. In our study, parameters of contrast agent application were not standardized and different interventional radiologists performed the study-interventions which may result in deviations in contrast application that influence the color-coded images. Nevertheless, this effect may be rated as minor, as in this study cohesive images, produced under the same conditions, were evaluated.

Second, the consensus was created by two of the interventional radiologists also participating in the evaluation team. In order to ensure more independence in future investigations, efforts should be made to have a consensus built by a group of radiologists that are not part of the evaluation team.

Third, because of the retrospective nature of this study, no objective parameters, like for example ankle-brachial-index (ABI), were investigated. Including those additional parameters may have increased the validity of consensus decisions and should be considered in coming investigations. Fourth, the current study addresses the question concerning superiority of one of the imaging techniques taken by itself. There was no evaluation of value of combining both imaging modes in comparison with conventional monochromatic DSA alone. Furthermore, one important variable that has been missing in the study was the time to reach the diagnosis and the ease of the diagnosis. The color coded DSA may not change the accuracy, but can be easier for interventional radiologists to read and help them to make their decisions faster. Since these topics have not been addressed in our study, subsequent studies are desirable.

## Conclusions

Color-coding DSA is a feasible and fast post-processing method which did not provide additional benefit in the evaluation of necessary stent implantations in bailout situations in this study. However, based on this cross-sectional study alone our results should be interpreted with care. Referring to this, the color-coded technology requires further investigation also addressing the time requirement for each DSA mode and taking a regular training of interpreters into consideration.

## Supplementary information

**Additional file 1.**

## Data Availability

The date used and analyzed during the current study are available on reasonable request.

## Abbreviations

ABI: Ankle-brachial-index
AUC: Area under the curve
DSA: Digital subtraction angiography
PTA: Percutanous transluminal angioplasty
SFA: Superficial femoral artery
PACS: Picture archiving and communication system
PAOD: Periphere arterial occusion disease
POBA: Plain old balloon angioplasty
PSVR: Peak systolic velocity ratio
ROC: Receiver Operating Characteristics
TTP: Time to peak
