# Supplementary material for: Evaluation of superficial femoral artery-lesions after percutaneous transluminal angioplasty: color-coded summation images vs. monochromatic digital subtraction angiography
Source: BMC Med Imaging. 2020 Jun 18;20:67. doi: 10.1186/s12880-020-00468-1 (PMC7302343; doi:10.1186/s12880-020-00468-1)
Supplement: Supplementary file 1 — Additional file 1. [file 12880_2020_468_MOESM1_ESM.pdf]

Julius-Maximilians-
UNIVERSITÄT
WÜRZBURG

Ethik-Kommission
bei der Medizinischen Fakultät

Ethik-Kommission
Institut für Pharmakologie und Toxikologie
Versbacher Str. 9
97078 Würzburg

Vorsitzende: Prof. Dr. E.-B. Bröcker
Geschäftsführer: Dr. R. Wölfel
Sekretariat: S. Schmidt/M. Keidel/M. Geiger
Telefon 0049 (0)931 31 48315
Telefax 0049 (0)931 31 87520
ethikkommission@uni-wuerzburg.de
www.ethik-kommission.medizin.uni-wuerzburg.de

Ethik-Kommission • Versbacher Str. 9 • 97078 Würzburg

Frau Irina Werner

Würzburg, 27.06.2016

bei Schriftwechsel bitte angeben: 20160514 01

Beratung - Doktorarbeiten mit retrospekt. Pt-Daten

Projekt: Anwendung des IFLOW Algorithmus bei AVK der Arteria femoralis superficialis.

Sehr geehrte Frau Werner,

zu Ihrer Anfrage vom 10.04.2016 zur retrospektiven Auswertung von Patienten- oder Untersuchungsdaten und deren Verwendung in Promotionsarbeiten oder in Publikationen nimmt die Ethik-Kommission wie folgt Stellung.

Grundsätzlich gilt bei einer Auswertung von bereits vorhandenen, klinikinternen anonymisierten Routinedaten bzw. Daten von individuellen Heilversuchen, dass keine Beratung durch die oder eine Antragstellung bei der Ethik-Kommission nach geltendem Recht erforderlich ist.

Es obliegt dem verantwortlichen Untersucher dafür Sorge zu tragen, dass lediglich Daten in die Auswertung einfließen, die unter Beachtung einschlägiger rechtlicher Vorgaben als auch berufsethischer Aspekte generiert wurden und dass geltende Datenschutzbestimmungen eingehalten werden.

Mit freundlichen Grüßen

Prof. Dr. med. Eva-Bettina Bröcker
Seniorprofessorin
Vorsitzende der Ethik-Kommission

Ausgefertigt im Auftrag

Dr. med. Reinhard Wölfel
Geschäftsführer der Ethik-Kommission
